# Coupling and Regulation of Porous Carriers Using Plasma and Amination to Improve the Catalytic Performance of Glucose Oxidase and Catalase

**DOI:** 10.3389/fbioe.2019.00426

**Published:** 2019-12-13

**Authors:** Lingtong Liao, Yuling Meng, Ruiming Wang, Baolei Jia, Piwu Li

**Affiliations:** ^1^School of Bioengineering, Qilu University of Technology (Shandong Academy of Sciences), Jinan, China; ^2^State Key Laboratory of Biobased Material & Green Papermaking, Qilu University of Technology (Shandong Academy of Sciences), Jinan, China

**Keywords:** poly glycidyl methacrylate, co-immobilization, plasma, glucose oxidase, catalase

## Abstract

Multiple enzyme systems are being increasingly used for their high-efficiency and co-immobilization is a key technology to lower the cost and improve the stability of enzymes. In this study, poly glycidyl methacrylate (PGMA) spheres were synthesized using suspension polymerization, and were used as a support to co-immobilize glucose oxidase (GOx) and catalase (CAT). Surface modification was carried out via a combination of plasma and amination to promote the properties of the catalyzer. The co-immobilized enzymes showed a more extensive range of optimum pH and temperature from 5.5 to 7.5 and 25 to 40°C, respectively, compared to free enzymes. Furthermore, the maximum activity and protein adsorption quantity of the co-immobilized enzymes reached 25.98 U/g and 6.07 mg/g, respectively. The enzymatic activity of the co-immobilized enzymes was maintained at ~70% after storage for 5 days and at 82% after three consecutive cycles, indicating that the immobilized material could be applied industrially.

## Introduction

Immobilizing biocatalysts has been of interest for the higher resulting activities and stabilities than those of free enzymes in biotechnological and industrial applications (Bilal et al., 2019). Presently, multiple enzymes are used to manufacture more products (Touahar et al., 2014; Ren et al., 2019). Co-immobilized enzymes could address the disadvantages of high-cost, non-recyclable nature, and low stability of free enzymes (Cui et al., 2017). Therefore, tethering multi-enzymes on a solid scaffold to facilitate biotransformation in a cell-free manner is a developmental strategy (Khattak et al., 2014; Schmid-Dannert and Lopez-Gallego, 2019). Gu et al. (2019) co-immobilized laccase and 2,2′-azino-bis(3-ethylbenzothiazoline-6-sulphonic acid) (ABTS) to obtain an efficient beaded biocatalyst for the degradation of indole, where ABTS improved the catalytic performance of laccase by promoting transfer of electrons or hydrogen atoms in the enzymatic redox reaction. Gao et al. (2019) co-immobilized chloroperoxidase (CPO) and glucose peroxidase on a titanium dioxide (TiO_2_) thin film for cascaded decolorization of Orange G dye. In this system, the hydrogen peroxide (H_2_O_2_) generated *in situ* by glucose peroxidase was directly utilized by CPO.

The porous solid material poly glycidyl methacrylate (PGMA), which was chosen as an optimal candidate for co-immobilizing GOx and CAT in this study, provides a suitable platform for consecutive reactions thus improving the reaction rate of enzymes (Jia et al., 2014). Particularly, the particle and pore sizes of the carrier are important factors affecting the catalytic efficiency of the immobilized enzyme. In addition, PGMA exhibited suitable physical properties such as hardness and controllable particle size, which were conducive to the recovery of enzymes, with a pore size that can be adjusted by changing the synthesis method and conditions (Li et al., 2014). Wang et al. (2018) fabricated three monodispersed PGMA/polystyrene-based spheres as carriers to achieve saturation adsorption capacity and selective adsorption of bovine serum albumin (BSA). The protrusions, holes, and pore canals of the spheres increased their specific surface area and overcame the diffusional limitations. Huang et al. (2018) synthesized porous poly (styrene-divinylbenzene) resin carriers with a 30 nm pore size and 72 m^2^/g specific surface areas, with an equilibrium adsorption amount of nuclease P1 of 4.02 mg/g. In addition, the low toxicity, less waste generation, and low-cost factors also met the needs of industrial production.

Carrier surface properties play a vital role in enzymatic reactions between substrates and biocatalysts. Surface modification is an effective strategy to improve surface properties by introducing various surface functionalities (Talbert and Goddard, 2012; Rodrigues et al., 2013). Low temperature plasma treatment technology is the preferred method of surface treatment over other traditional modifications because of its specific qualities such as lower operating costs, no discharge of additional waste, clean operating processes, and high operational safety (Cheng et al., 2014; Minati et al., 2017). The molecules or ions in the reaction chamber collide to form a plasma flow under special conditions, are highly active with sufficient energy to form multiple chemical bonds, and possess many polar functional groups on their exposed carrier surfaces. Wu et al. (2019) treated corn starch with an atmospheric pressure plasma jet (30 min), which increased the solubility and paste clarity of the ingredients to meet the food application needs. Kahoush et al. (2019) treated virgin carbon felt with cold remote plasma to increase the wettability of non-woven carbon fiber felts.

For the immobilization process, modified PGMA with versatile epoxy groups could cross-link with enzyme to form the so-called short spacer arm, aldehyde-ammonia bond, in the presence of glutaraldehyde (GA). This immobilization method is similar to that reported in previous articles (Du et al., 2009; Nguyen et al., 2019). To the best of our knowledge, the enzyme activities of GOx/CAT on aminated PGMA with surface modifications induced by plasma treatment have not been investigated.

**Figure 1:**
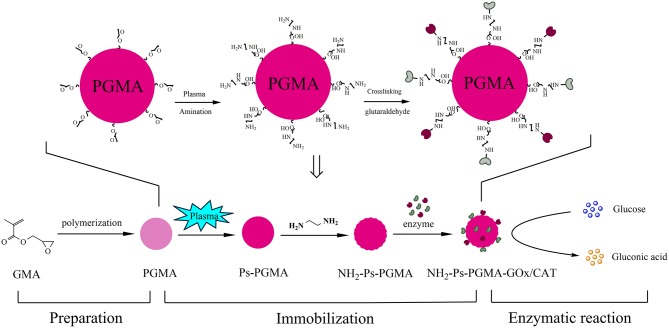
The complete schematic design of the immobilization process.

The combination of GOx and CAT, allows GOx to catalyze the oxidation of glucose to produce gluconic acid and H_2_O_2_ (in the presence of oxygen as an electron acceptor), which then simultaneously inactivates GOx. Addition of CAT could catalyze the conversion of H_2_O_2_ to water and oxygen (Oliveira Mafra et al., 2019). Co-immobilization of GOx and CAT could provide an effective strategy to achieve this cascade reaction. In this study, PGMA was used as a support and was synthesized from glycidyl methacrylate (GMA) using suspension polymerization in our laboratory. The effect of preparation conditions on the size and morphological of the spheres was investigated, and different rotational speeds and formulations of porogens were used systematically. Spheres with optimal reaction conditions were selected to immobilize the biocatalyst. Plasma treatment was combined with surface treatment of samples to improve the material properties and immobilization effects. Furthermore, the reusability and storage stability of the co-immobilized enzymes were analyzed. The complete schematic design of the immobilization process is shown in Figure 1.

## Materials and Methods

### Materials and Chemicals

GMA and ethylenedimethacrylate (EDMA) were purchased from Nine-Dinn Chemistry, Co., Ltd. (99%, Shanghai, China). Polyvinyl alcohol (PVA) and 2,2-azobisisobutyronitrile (AIBN) were purchased from Damao Chemical Reagent Factory (Tianjin, China). 1,3,5-Trially-1,3,5-triazine-2,4,6-trione (TAIC) was purchased from HaoHong Biomedical Technology Co., Ltd. (Shanghai, China). GA (AR, 50%) was commercially obtained from Rhawn Chemical Technology Co., Ltd. (Shanghai, China). Ethylenediamine was purchased from Taicang Hushi Reagent Co., Ltd. (Shanghai, China). Nano-calcium carbonate (CaCO_3_) was obtained from Yuanye Bio-Technology Co., Ltd. (70–100 nm, 99% purity, Shanghai, China). Toluene and normal heptane were purchased from Fuyu Fine Chemical Co., Ltd. (Tianjin, China). GOx (6,500 U/g) and CAT (50,000 U/g) were provided by Novozymes Biotechnology Co., Ltd. (Nanjing, China).

### Preparation of Poly Glycidyl Methacrylate Spheres

PGMA spheres were prepared using modified suspension polymerization as previously reported (Arica et al., 2009; Oh et al., 2010). Briefly, a 250 mL flask with three necks was used for the preparation with 6.83 mL GMA as the crosslinking monomer in the presence of 2.74 mL TAIC and 2.5 mL EDMA as the crosslinker, an aqueous solution of PVA (1% [v/v]) as the dispersant, and 7.77 mL toluene and 0.01 g nano-CaCO_3_ as the porogen. The polymerization reaction was performed with continuous stirring for 2 h at 75°C after adding 2% AIBN as an initiator under a nitrogen atmosphere, and the mixture was then heated at 85°C for 2 h. The obtained spheres were indurated for 24 h in absolute ethanol, washed with distilled water followed by vacuum freeze-drying, and were finally stored at 4°C for further use.

### Modification of Spheres Using Amination/Plasma Treatment

**Figure 2:**
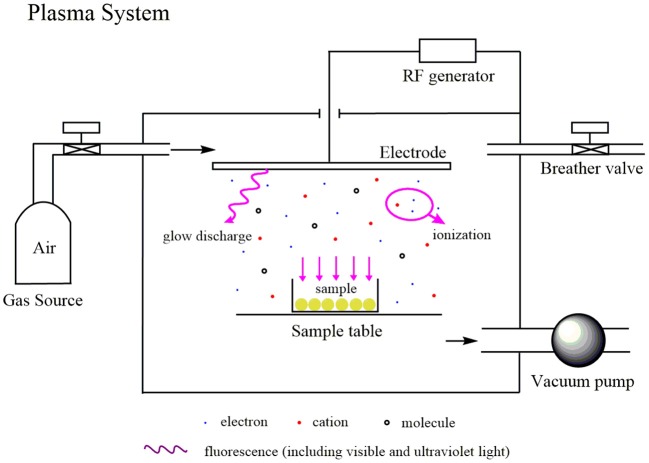
The process of PGMA modification by the plasma system.

The initial porous spheres (0.25 g) were packed in a glass container covered with two-layer gauze and were placed in a reaction chamber filled with air at −98.0 kPa under vacuum evacuation. Then, the carriers were first treated with the output of the radio frequency (RF) power supply, followed by air plasma operated at 40 kHz and were delivered at 80 W for 60 s, with an airflow rate set to 200 mL/min. This process was repeated three times, ensuring that the samples were rotated to treat all sides of the spheres as equally as possible. A sketch of the plasma system is shown in Figure 2.

The plasma power modification was performed as follows: carriers of the same mass were treated with different plasma powers to determine the optimum power of plasma treatment for enzymatic activity improvement.

The PGMA and activated Ps-PGMA were suspended in 2% (v/v) ethylenediamine solution while stirring for 4 h at 60°C, followed by continuous washing with 0.8 M phosphate-buffered saline (PBS, pH 7) until no ethylenediamine was left on the carrier. The obtained material was designated as NH_2_-PGMA and NH_2_-Ps-PGMA and stored at 4°C.

Four types of the modification process sequence were discussed: (a) PGMA were treated with the plasma system or (b) ethylenediamine solution separately; (c) PGMA were immersed in the ethylenediamine solution followed by plasma treatment; (d) PGMA were modified by plasma and then treated with ethylenediamine solution.

### Preparation and Characterization of Co-immobilized Enzymes

GOx and CAT were diluted with 0.8 M phosphate buffer, 0.2% (w/v) 1-ethyl-3-(3-dimethylaminopropyl)carbodiimide (EDC) and 0.2% (w/v) N-hydroxysuccinimide (NHS) were added to the enzyme solution, and the mixture was kept for 30 min at 4°C. Then, 0.25 g PGMA was added to the mixed enzyme solution for 15 min, followed by 1% GA for crosslinking for 1.5 h. Afterwards, 5 mL saturated ammonium sulfate was added to the mixture while vibrating at 30°C for 1 h, followed by washing with 0.8 M phosphate buffer (pH 7) to remove the unbound or loosely bound enzyme. The supporter co-immobilized with the enzymes was named NH_2_-Ps-PGMA-GOx/CAT.

Fourier transform infrared (FTIR) spectroscopy was conducted using a Thermo Nicolet 10 FTIR instrument (China) to determine the varied surface chemical groups of the PGMA, Ps-PGMA, and NH_2_-Ps-PGMA as well as NH_2_-Ps-PGMA-GOx/CAT. All the dried samples were recorded within 400–4,000 cm^−1^ with a resolution of 2 cm^−1^.

Samples were characterized using scanning electron microscopy (SEM) with the Regulus 8220 SEM system (China) after sputter-coating with an ultrathin layer of gold to examine the external and internal morphology of the spheres.

### Assay of Co-immobilized and Free Enzyme Activity

Determination of the free enzyme activity of the mixture: A standard glucose solution (pH 6) with a concentration of 5 mg/mL was prepared and a dilution of 1 mL GOx and CAT solution was added to 5 mL of the glucose solution. After reacting at 30°C for 10 min, the enzymes were immediately inactivated at 100°C, the mixture was centrifuged for 5 min, the supernatant was collected and diluted properly, and the residual glucose concentration was determined using the SBA-40D biosensor analyzer (Biology Institute of Shandong Academy of Sciences, China).

Assay of immobilized enzyme activity (Mahdizadeh and Eskandarian, 2014): Briefly, 0.25 g of the carrier was mixed with 5 mL of the standard glucose solution (pH 7). The reaction was allowed to run at 35°C for 10 min and then the residual reducing glucose concentration was measured using the SBA biosensor analyzer after diluting appropriately.

One unit of the immobilized enzyme was defined as the amount of enzyme that catalyzed the oxidation of 1 μmol glucose/min at 35°C and pH 7.

The GOx activity was calculated using the following equation:

$$\begin{array}{l} GOD\;activity\left(U.mL^{-1}\right)\;=\left(C_{o}-C\right)\times V\times f/10 \end{array}$$

where, *C*_0_ is the initial glucose concentration detected at the optimal pH and temperature (mg·mL^−1^), c is the final glucose concentration of the sample detected under the same conditions (mg·mL^−1^), V is the volume of the reaction system solution of the substrate and enzyme, f represents the dilution multiples, and 10 is the reaction time (min).

The maximum consumption of the substrate was considered to be 100%, and then the relative activity was calculated as follows:

$$\begin{array}{l} Relative\;activity\left(\%\right)\\ =enzymatic\;activity/maximum\;enzymatic\;activity\times 100\% \end{array}$$

### Optimum Temperature and pH of Free and Co-immobilized Enzymes

The effect of temperature on the free and co-immobilized enzymes was investigated by hydrolyzing 5 mg/mL glucose in phosphate buffer at various temperatures from 25 to 45°C for 10 min.

The optimal pH was determined based on the activities of free and co-immobilized enzymes over a range from pH 5.0 to 8.0 for 10 min at 35°C.

### Kinetics of Free and Immobilized Enzyme Activity

The enzyme kinetic activity was determined by adding 1 mL properly diluted free enzyme and 0.25 g immobilized enzyme to 10 mL glucose solution at concentrations ranging from 0 to 5 mg/mL. The reaction was controlled for exactly 10 min with stirring to determine the initial reaction rate and then the Michaelis-Menten constant (*K*_m_) and maximum reaction velocity (*V*_max_) were calculated using the Lineweaver-Burk analysis.

### Reusability and Stability of Co-immobilized Enzymes

The enzymatic activity assay was conducted in multiple cycles for each sample as described in section Assay of Co-immobilized and Free Enzyme Activity. This procedure was conducted in 7 reaction cycles (10 min each) during which 5 mg/mL glucose solution was oxidized under optimal conditions. After each cycle, the catalyst spheres were collected and washed three times with phosphoric acid buffer (pH 7) to remove the residual substrate that adhered to the surface of the support material, which was then reintroduced into the next cycles.

In the storage stability study, the activity of the co-immobilized enzymes was measured for 10 days after each experimental cycle, and the co-immobilized enzymes were stored at 4°C.

## Results and Discussion

### Carrier Optimization and Characterization

The specific surface area and interior pore size of the spheres are both important in enzyme immobilization and should not be considered negligible in designing the skeletal structure of the immobilized material. The specific surface area of the spheres is determined by the size of the PGMA spheres and therefore, spheres were synthesized at various rotational speeds (Hu et al., 2019). The sizes of the supporters varied as the rotational speed changed (200, 350, 500, and 650 rpm) (Figure S1). The spheres synthesized at 650 rpm were too small to be recovered and difficult to formulate into regular spheres. This observation indicated that the porous material should be of a suitable size, which depended on using an optimum speed (500 rpm).

The surface and internal pore sizes of the spheres play a vital role in effective binding of the enzyme proteins to the material, and diverse porogens would eventually affect the pore sizes of the material (Miao et al., 2019). In this study, the supports were prepared with different types of porogens (Figure S2). The surface of the supports consisted of a single liquid porogen (toluene), which presented as an irregular aperture that may be related to its properties (Torres et al., 2008). During polymerization, part of the pore collapsed because of the change in reaction temperature, resulting in the volatilization of the porogen before the carrier had hardened. The use of a toluene/heptane mixture as the porogen would cause fragmentation instead of formation of complete spheres. When a solid-liquid porogen was used, the nano-CaCO_3_ adequately retained the pore shape.

**Figure 3:**
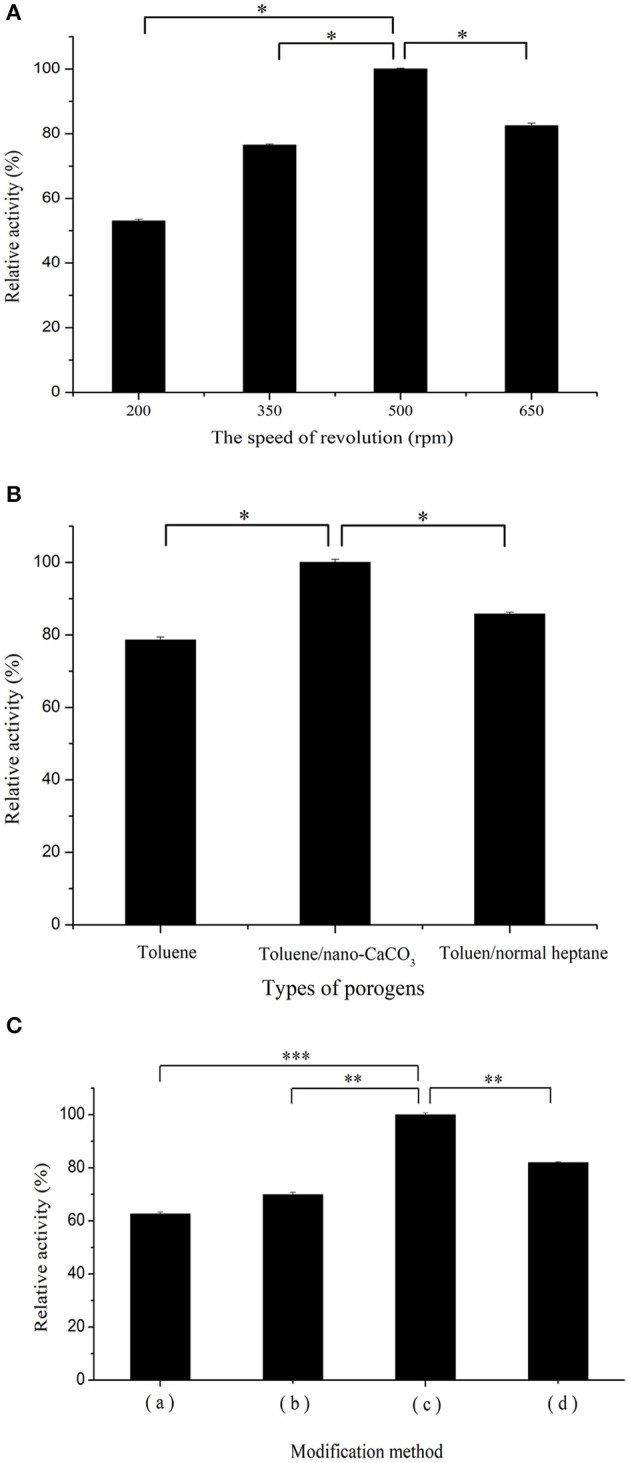
**(A)** Effect of rotational speed: variations of particle size and morphologies of spheres prepared using four rotational speeds: (a) 200, (b) 350, (c) 500, and (d) 650 rpm. **(B)** Effect of three porogen formulas: (a) single liquid (toluene only), (b) mixed liquid (toluene/heptane), and (c) solid-liquid porogen (toluene/nano-calcium carbonate [CaCO3]). **(C)** Surface modification protocol on relative activity of co-immobilized enzymes: (a) PGMA were treated with the plasma system; (b) PGMA were treated with the ethylenediamine solution; (c) PGMA were immersed in the ethylenediamine solution followed by plasma treatment; (d) PGMA were modified by plasma and then treated with ethylenediamine solution. All the activity measures were repeated thrice to ensure accuracy. *n* = 3, *bars* (*P < 0.05; **P < 0.01; ***P < 0.001).

To investigate the effect of sphere size on co-immobilization of the two enzymes, the activity of co-immobilized enzymes on spheres prepared at different rotational speeds was determined in Figure 3A. In contrast to spheres with a medium diameter, larger-sized spheres possessed a larger pore volume, which made it easier for the enzyme to be lost under harsh environments. Small diameter spheres with pores that were too small formed extremely complicated channels, which impeded contact between the enzymes and substrates, consequently decreasing the relative activity. In addition, excessive rotational speed could result in fragmentation of spheres and decreased enzyme activity (Mouarrawis et al., 2018).

The effect of various porous frameworks prepared from different porogens on the immobilization was studied. As shown in Figure 3B, the carrier composed of a solid-liquid porogen (toluene and nano-CaCO_3_ 0.05 g) clearly exhibited better relative activity than that composed of the single liquid (toluene only) and mixed liquid (toluene/heptane); the latter leads to fragmentation of spheres. Compared to spheres prepared with liquid porogen, the increased surface area of spheres formulated with the solid-liquid porogen contributed to improving enzyme adsorption capacity, leading to higher enzymatic activity. Optimized PGMA that provides appropriate positions and distances for co-immobilized catalysts is expected to promote the overall cascade process by regulating porosity (Bolivar et al., 2011).

### Surface Modification of Carrier Using Plasma/Amination Treatment

Effective binding of porous materials and enzymes is impossible by direct adsorption only and consequently, modification of carriers is indispensable to this process. There are abundant epoxy groups on the surface of the supporter, which react directly with amino, hydroxyl, and thiol groups (Rodrigues et al., 2014). In this study, amination and plasma treatment were chosen as modification methods before immobilization. Earlier studies have shown that amination can be used to convert epoxy groups into amino groups (Rodrigues et al., 2011). Furthermore, plasma treatment was used to facilitate the electrostatic attraction and randomly increase the number of various groups on the carrier surface (Vasiliev et al., 2019). The effect of using amination or plasma alone is obviously weaker, and the surface properties of the carrier were improved by the combined use of plasma and ethylenediamine. Plasma treatment has the advantage of introducing various groups randomly. Consequently, performing plasma treatment first, followed by ethylenediamine may allow more effective groups induced by plasma to convert into amino groups, thereby increasing the modification effect. In Figure 3C, experimental analysis of the treatment sequence revealed that the treatment showing a higher relative activity was plasma treatment followed by amination. To optimize the plasma treatment method, plasma power as an important factor affecting enzyme activity was used based on a previous report (Martins et al., 2009) and consequently, the activity of the immobilized enzyme was enhanced by changing the plasma strength. Figure S3A shows the trend of enzyme activity alteration with different plasma power treatments. Increasing the power of plasma treatment promoted the formation of active sites on the carrier surface. The probability of carriers combined with the enzymes was increased, which enhanced the enzymatic activity. However, further increase in power caused the enzymatic activity to reach a plateau. We presumed that the active groups were saturated, or the spatial location was limited, causing the enzymatic activity to show a tendency to stabilize.

Compared with the traditional surface optimization method, the plasma technology used in this work was cleaner and convenient. The carrier modified by plasma or amination alone has similar morphology and chemical properties compared with the previous experimental results. This work has combined the use of two methods, and the effect of the immobilized enzyme has been improved. In future experiments, we will investigate the effect of enzyme immobilization of various materials under the combined treatment, and find a general mechanism to explain the immobilization process.

### Optimization of Immobilization Conditions and Characterization

After surface modification, optimization of the immobilization process is essential to obtain the best immobilization efficiency (Schoffelen and van Hest, 2012). GA acts as a bi-functional cross linker that reacts with amino groups of enzymes and activated carriers (Barbosa et al., 2014). The main reaction in the immobilization process is the GA/amino reaction and therefore, the effects of a series of GA concentrations, 0.25–2.5%, on immobilization were examined. The results showed that the highest enzymatic activity was achieved when the concentration reached 1%, and these results are shown in Figure S3B.

**Figure 4:**
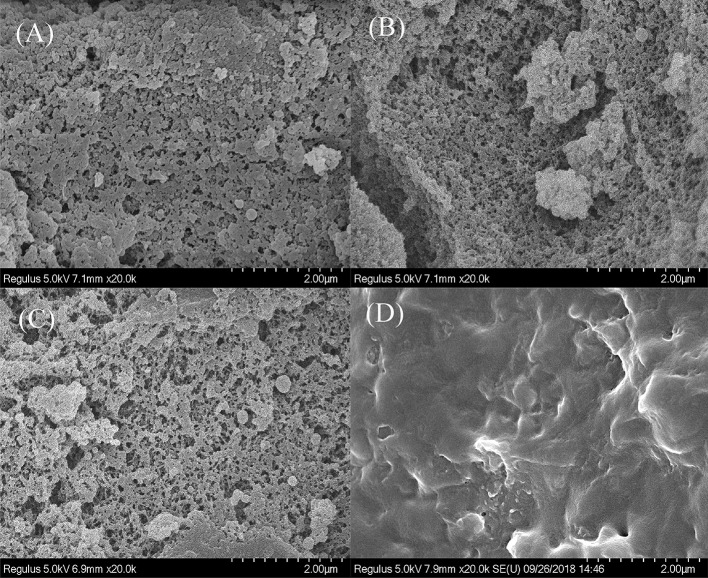
Scanning electron microscopy (SEM) images of sphere surfaces. **(A)** PGMA: initial poly glycidyl methacrylate (PGMA) sphere, **(B)** Ps-PGMA: plasma-treated (activated) PGMA sphere **(C)** NH_2_-Ps-PGMA: activated Ps-PGMA sphere subjected to amination treatment, and **(D)** NH_2_-Ps-PGMA-GOx/CAT: NH_2_-Ps-PGMA sphere immobilized with glucosidase (GOx) and catalase (CAT).

To verify that the enzymes were successfully immobilized on the modified carrier, SEM and FTIR spectroscopy were used and the morphologies of PGMA, Ps-PGMA, NH_2_-Ps-PGMA, and NH_2_-Ps-PGMA-GOx/CAT are presented in Figures 4A–D. Initially, the surface of unmodified PGMA, which had a porous structure with numerous cavities, became smoother following the use of the plasma systems. After plasma treatment, a layered structure with wrinkles was clearly observed on Ps-PGMA, indicating that the plasma subsequently produced conformal coatings on the surface of PGMA, which were in accordance with those reported by another study (Bu et al., 2019). The results suggest that the physical morphology and chemical groups were changed by plasma treatment (Mostofi Sarkari et al., 2019). After amination, the relatively higher amount of functional (mostly amino) groups of NH_2_-Ps-PGMA provided an appropriate size of the environment for co-immobilizing GOx/CAT, although the differences in images were not obvious after the amination process. Finally, the images of NH_2_-Ps-PGMA-GOx/CAT showed that the spatial position of the pore structure, which had been invisible after the multistep modification processes, was occupied by functional groups and enzymes. In addition, the decrease in surface roughness might verify that the enzymes were successfully immobilized on the modified PGMA.

**Figure 5:**
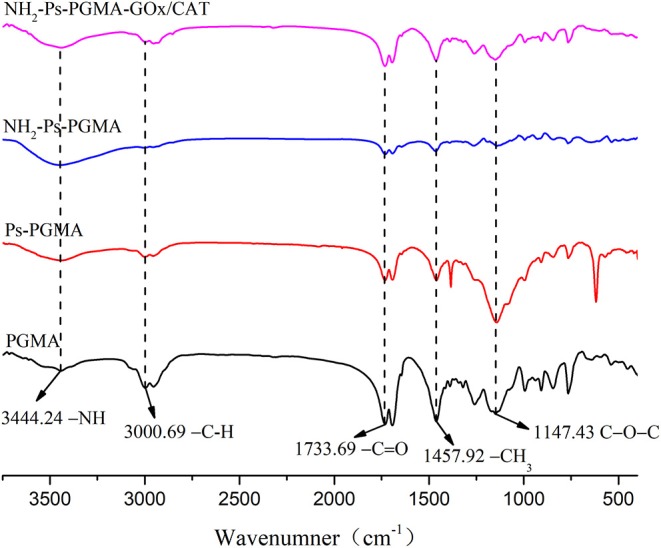
Fourier transform infrared (FTIR) spectra of PGMA, Ps-PGMA, NH_2_-Ps-PGMA, and NH_2_-Ps-PGMA-GOx/CAT.

FTIR spectroscopy was performed to corroborate the chemical bonding behaviors of the immobilization process. For Figure 5, the spectrum was partly similar to that reported previously (Bayramoglu et al., 2011; Zhuang et al., 2019) on account of the homologous materials, but the plasma treatment caused discrepancies. The IR spectra of the four samples (PGMA, Ps-PGMA, NH_2_-Ps-PGMA, and NH_2_-Ps-PGMA-GOx/CAT) showed the vibrational frequencies and size of the characteristic peak in each processing stage of the immobilization process, indicating that chemical interactions likely occurred on the surface of these materials during the carrier modification and co-immobilization processes. The sharp, intense characteristic peak of the original material at 1,147 and 1,733 cm^−1^ indicated the stretching vibrations of epoxy C-O-C and carboxyl C=O, respectively, suggesting the presence of abundant oxygen-containing functional groups in PGMA. The spectra confirmed the integration of new functional groups on the surface of Ps-PGMA. The peaks were partly strengthened, and some new peaks appeared concurrently, which demonstrated surface modification by the air plasma treatment. A significant reduction in the characteristic peaks of -C=O and C-O-C suggested the introduction of amino groups on NH_2_-Ps-PGMA following ethylenediamine treatment. The reduction of adsorption peaks of -NH_2_ at 3,444 cm^−1^ and -C=O at 1,733 cm^−1^ indicated the successful introduction of a short spacer arm by the achievement of an intense multipoint covalent attachment between an enzyme molecule and a rigid support.

### Optimum Catalytic Conditions for Free and Co-immobilized Enzymes

**Figure 6:**
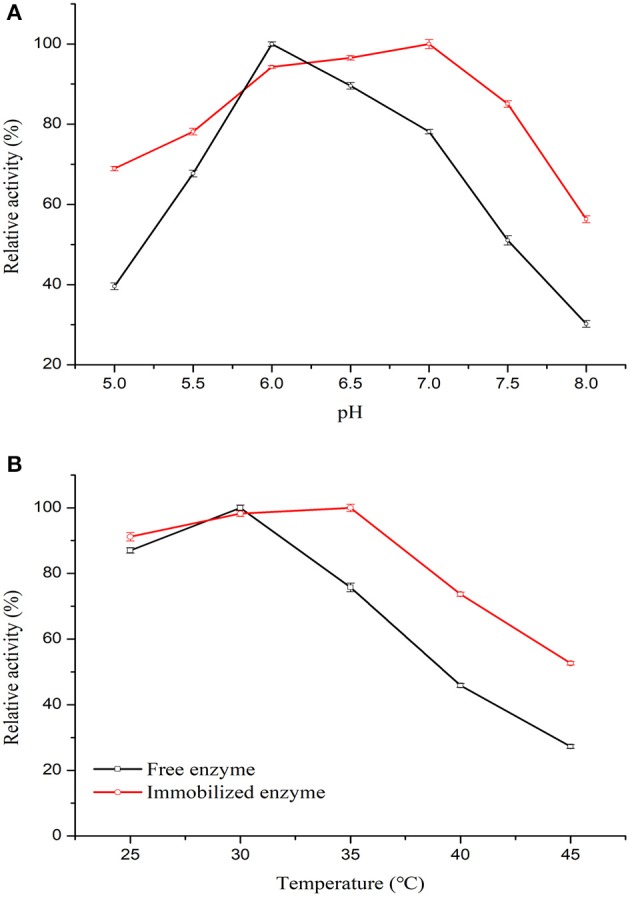
Properties of free and co-immobilized enzymes. **(A)** The effect of temperature on the free and co-immobilized enzymes was investigated from pH 5.0 to 8.0 for 10 min at 35°C. **(B)** The effect of temperature on the free and co-immobilized enzymes was investigated from 25 to 45°C for 10 min.

To elucidate the effect of acid-alkali tolerance of free and immobilized enzymes on enzymatic catalysis, the optimum pH was determined. The results in Figure 6A show that after covalent crosslinking on the carrier, the co-immobilized enzymes exhibited higher relative activity than that of the free enzymes at pH 5.5–7.5. This observation revealed that the pH stability of the enzymes was enhanced after co-immobilization on PGMA. The optimum pH for the free and co-immobilized enzymes would positively affect the spatial structure of the enzymes by changing the conformation of the active site and the ionic form of functional groups (Li et al., 2018).

To investigate the effect of temperature on enzyme stability, their catalytic activity at different temperatures was tested (Vardar et al., 2018). Figure 6B shows the behavior of co-immobilized and free enzymes assessed from 25 to 45°C. The optimal temperature for free enzymes was in the range of 25–35°C, whereas that of the co-immobilized enzymes was broader at 25–40°C. The immobilization and modification process thus had a positive effect on enzyme stability. The improved thermal stability may be due to the multipoint interaction via amino groups and GA of the enzymes with the support material, which likely reduced the temperature-induced conformational changes of the enzymes, protecting them from inactivation (Huang et al., 2011). The results showed that GOx/CAT-PGMA possessed the highest enzymatic activity at pH 7.0 with an optimal temperature of 35°C.

### Enzyme Protein Adsorption and Kinetics

**Figure 7:**
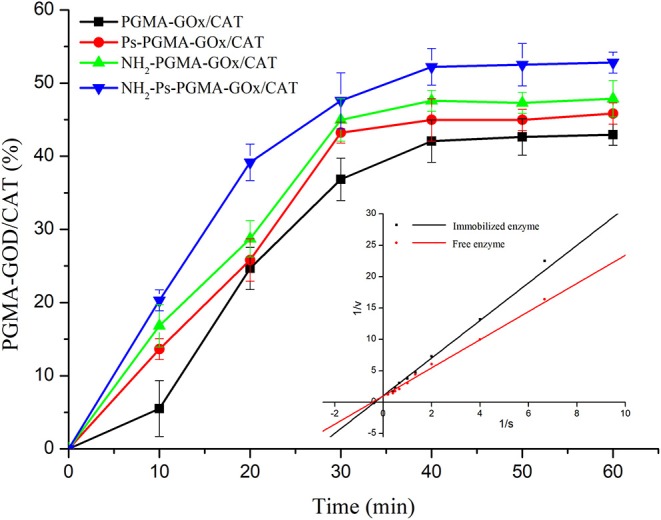
Adsorption capacity of poly glycidyl methacrylate (PGMA), plasma-treated PGMA (Ps-PGMA), aminated PGMA (NH_2_-PGMA), and plasma-treated and aminated PGMA (NH_2_-Ps-PGMA) in 1 h at 35°C and pH 7. Insert map shows catalytic kinetic plots of free and co-immobilized enzymes. All activity measurements were repeated thrice to ensure accuracy.

To study the adsorption capacity of enzymatic proteins on multi-hollow microspheres prepared using different treatment methods, a dynamics experiment was conducted. The adsorption capacity of PGMA, Ps-PGMA, NH_2_-PGMA, and NH_2_-Ps-PGMA under the same conditions is clearly shown in Figure 7. During the adsorption process, all the carriers showed varying adsorption capacities for the enzymes, and reached the adsorption equilibrium in approximately 40 min. The adsorption capacity of NH_2_-Ps-PGMA was clearly shown to be higher than that of the original PGMA carrier, achieving approximately 53% equilibrium adsorption capacity. Furthermore, the adsorption capacity of Ps-PGMA and NH_2_-PGMA carriers was higher than that of PGMA and, particularly, was further improved by the combination of plasma treatment and amination modification. This phenomenon can be explained by the enhanced adsorption ability of the porous material with a high surface area. Because of its chemical properties, the pore structure surface contains abundant active (including highly reactive epoxy) groups, which could subsequently bind enzyme protein by convenient conversion to groups such as hydroxyl, amine, and carboxyl (Kling and Ploehn, 1995). Therefore, plasma treatment and amination of the porous carrier generated more binding sites for the enzymes, thereby accelerating the adsorption efficiency and increasing adsorption capacity (Torres-Salas et al., 2011).

To compare the catalytic kinetic parameters of the free and co-immobilized enzymes, a kinetic experiment was performed. The *Vmax* of the co-immobilized enzymes (0.960 mM/[L·min]) was slightly lower than that of the free enzymes (1.018 mM/[L·min], insert chart in Figure 7). The *Km* of the co-immobilized enzymes (2.874 mM/L) was higher than that of the free enzymes (2.284 mM/L). The conformational changes of the enzyme molecules due to the covalent immobilization could reduce the accessibility of active sites of immobilized enzymes to the substrate molecules (Tse et al., 1987). The *Km* and *Vmax* of the enzymes differed little before and after immobilization. This observation could be attributable to the protection of enzyme activity of the co-immobilized enzyme by nanochannels on the surface and inside the spheres (Yu et al., 2014). Generally, the catalytic efficiency of the enzymes was maintained at a higher level after immobilization than that of the free enzymes. This observation suggests that the enzymes in the multi-enzyme system were in close proximity, enabling the creation of a microenvironment that promptly eliminated the by-products and mitigated the side effects of intermediates. In contrast to diffusion into an ambient environment, the product of the previous enzyme was directly transferred to the other as the substrate (Hwang and Lee, 2019). Therefore, the immobilization process used in this study achieved the goal of maintaining high enzyme catalytic efficiency.

### Reusability and Storage Stability of Co-immobilized Enzymes

**Figure 8:**
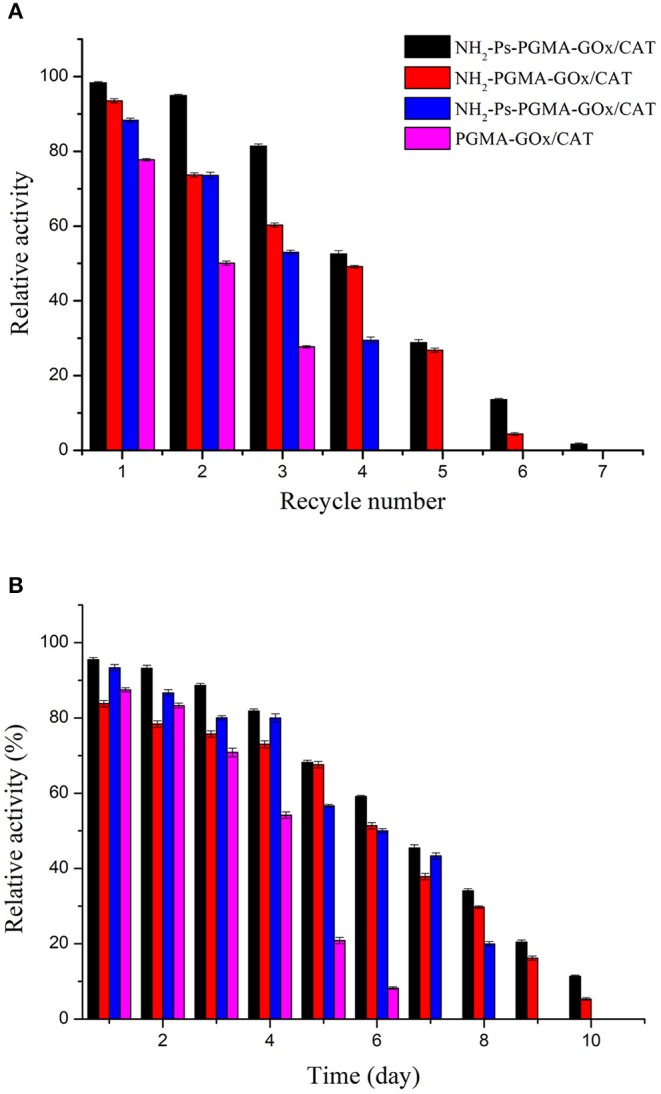
Reusability and storage stability of poly glycidyl methacrylate (PGMA), plasma-treated (activated) PGMA (Ps-PGMA), aminated PGMA (NH_2_-PGMA), and plasma-treated and aminated (NH_2_-Ps-PGMA) under optimum conditions: **(A)** the enzymatic activity assay was conducted for 7 reaction cycles under optimal conditions (35°C, pH 7), the carrier was recovered and washed three times with phosphoric acid buffer (pH 7) after each cycle, then reintroduced into the next cycles. **(B)** The catalytic activity was measured under optimal conditions (35°C, pH 7) for 10 days after each experimental cycle, and the catalyzer was stored at 4°C.

To validate the availability and effectiveness of the co-immobilized enzymes in practical operations, the recyclability and storage stability of the four co-immobilized enzyme systems were determined. The results of the reusability assessment are summarized in Figure 8A. After three consecutive cycles, NH_2_-Ps-PGMA-GOx/CAT maintained ~82% of its initial activity, which was superior to the performance of PGMA-GOx/CAT, indicating that combined use of both modification methods improved the rigidity of the enzyme proteins, preventing their dissociation (Fernandez-Lafuente, 2009). In addition, increasing the number of cycles rapidly decreased the activity of Ps-PGMA-GOx/CAT. This effect might have occurred because the random active groups introduced by plasma treatment were not as stable as the amino groups of NH_2_-PGMA-GOx/CAT, resulting in partial loss or inactivation of co-immobilized enzymes during recycling.

The storage stabilities of the co-immobilized enzymes were tested, and the result is shown in Figure 8B. The enzymatic activity was approximately 70% after storage for 5 days, which indicated good storage stability of the co-immobilized enzymes. Furthermore, Ps-PGMA-GOx/CAT exhibited better storage stability than that of NH_2_-PGMA-GOx/CAT, and NH_2_-Ps-PGMA-GOx/CAT exhibited the best stability. Mild treatment and use of fewer organic reagents reduced the activity damage to NH_2_-Ps-PGMA-GOx/CAT and extended the storage time of the enzymes (Siar et al., 2019).

## Conclusions

A novel co-immobilizing process for GOx/CAT was developed. PGMA was synthesized and modified using plasma/amination treatment under mild conditions, followed by process optimization. NH_2_-Ps-PGMA used for enzyme immobilization exhibited the maximum protein adsorption capacity and superior enzymatic activity compared to free enzymes and the other three formulations. NH_2_-Ps-PGMA-GOx/CAT showed similar affinity and catalytic rate for substrates to that of free enzymes, with a wider temperature and pH range, and superior storage stability, and reusability. The co-immobilized enzyme could be further applied for future production of gluconic acid, and this method could be used to develop a platform for co-immobilization of various enzymes in other industries.

## Data Availability Statement

All datasets generated for this study are included in the article/Supplementary Material.

## Author Contributions

PL and RW giving the idea for this project, helping in the case of scientific problems. LL was responsible for planning and performing experiments as well as writing the main part of the manuscript. YM was responsible for helping with experiments operation and data collation. BJ, PL, and LL were responsible for the final correction and English proofing.

### Conflict of Interest

The authors declare that the research was conducted in the absence of any commercial or financial relationships that could be construed as a potential conflict of interest.
