# Supplementary material for: Coupling and Regulation of Porous Carriers Using Plasma and Amination to Improve the Catalytic Performance of Glucose Oxidase and Catalase
Source: Front Bioeng Biotechnol. 2019 Dec 13;7:426. doi: 10.3389/fbioe.2019.00426 (PMC6923177; doi:10.3389/fbioe.2019.00426)
Supplement: Supplementary file 1 [file Data_Sheet_1.PDF]

Supplementary Material

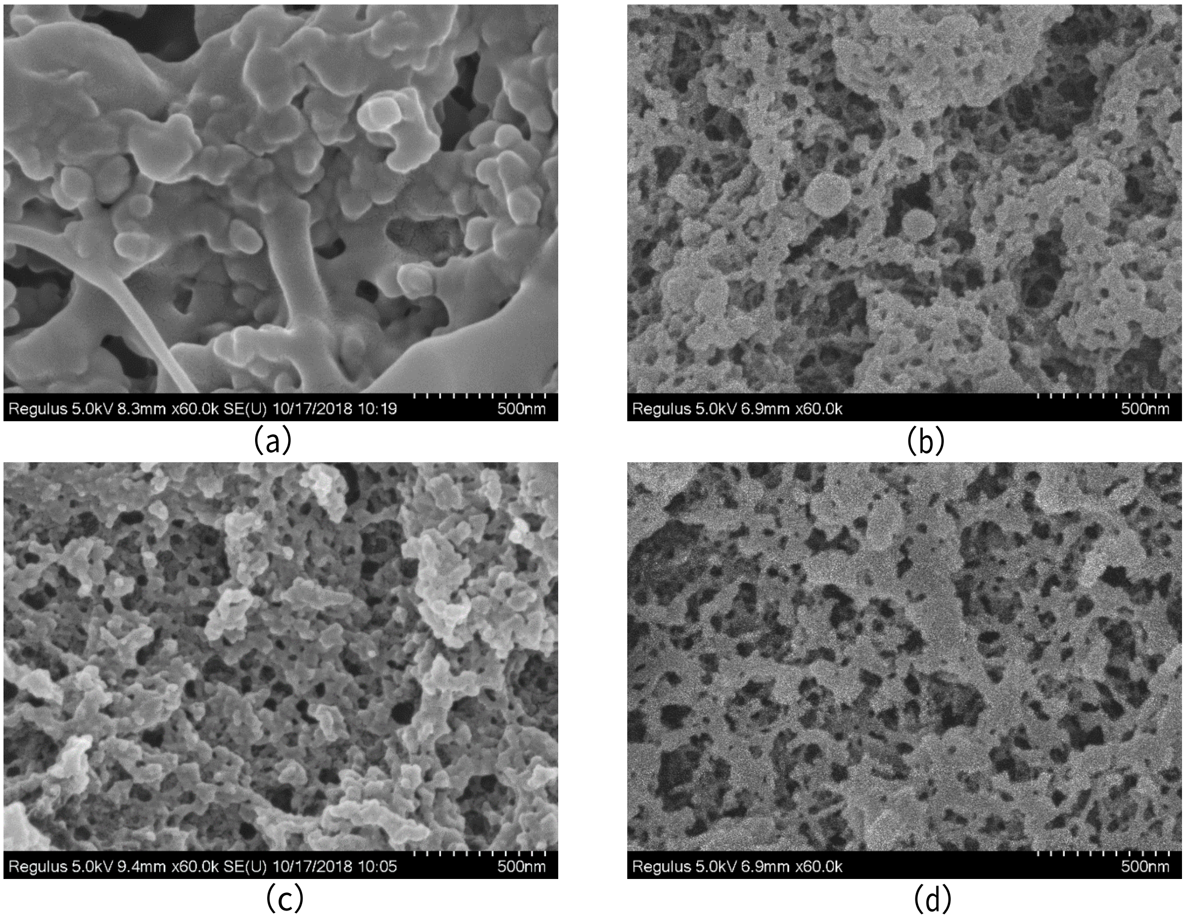

Supplementary Figure 1. Variations of particle size and morphologies of spheres prepared using four rotational speeds: (a) 200, (b) 350, (c) 500, and (d) 650 rpm.

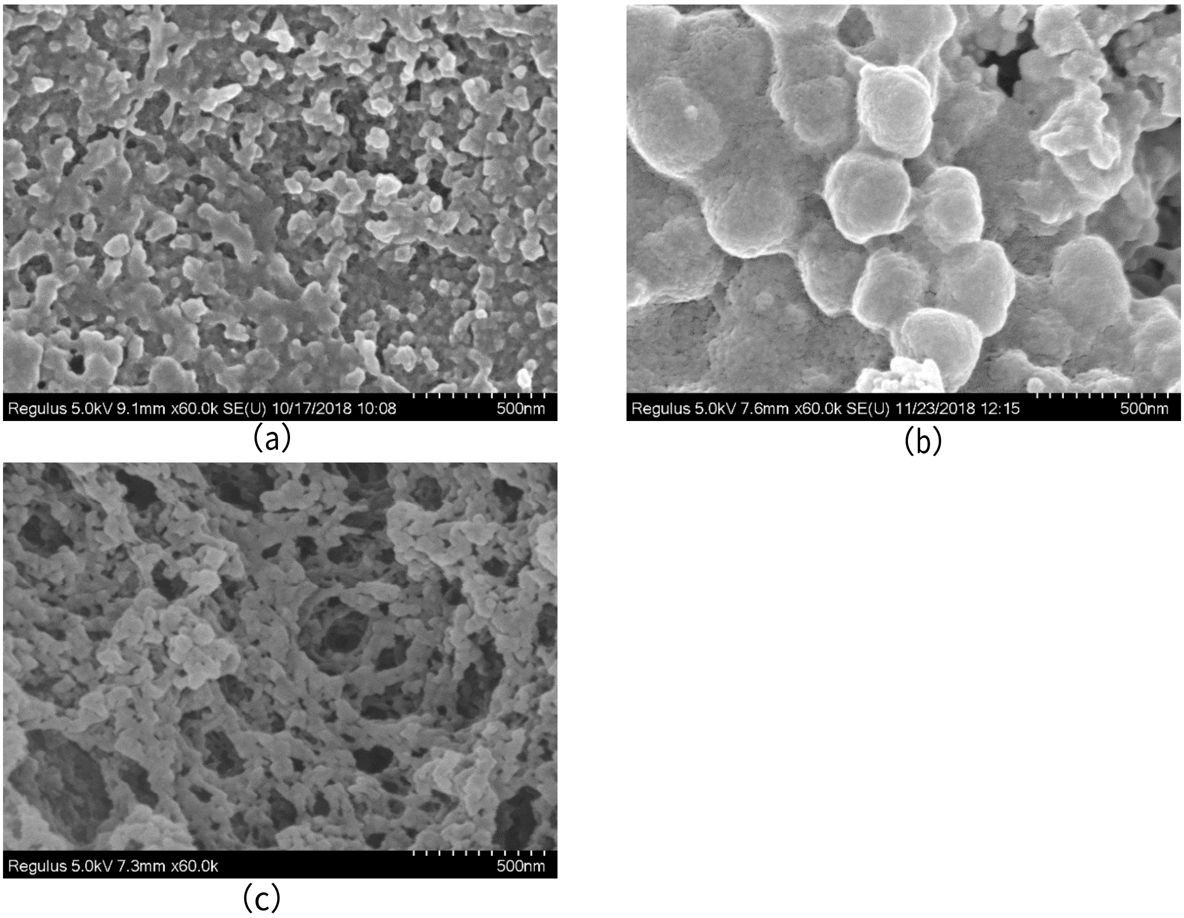

Supplementary Figure 2. Three porogen formulas: (a) single liquid (toluene only), (b) mixed liquid (toluene/heptane), and (c) solid-liquid porogen (toluene/nano-calcium carbonate [$CaCO_3$]).

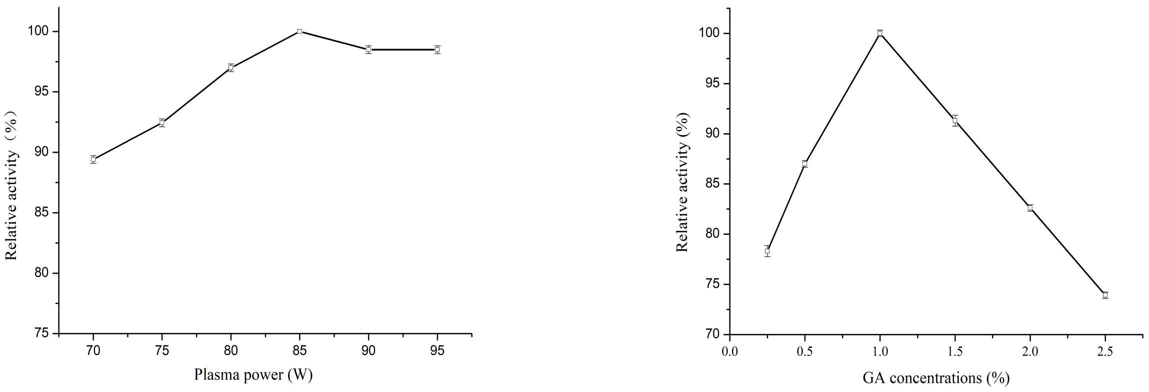

(a) (b)

Supplementary Figure 3. Effect of (a) Plasma power and (b) Glutaraldehyde concentration on relative activity of co-immobilized enzymes.
